# Supplementary material for: Advancements in monitoring: a comparison of traditional and application-based tools for measuring outdoor recreation
Source: PeerJ. 2024 Sep 10;12:e17744. doi: 10.7717/peerj.17744 (PMC11397128; doi:10.7717/peerj.17744)
Supplement: Supplemental Information 2 [file peerj-12-17744-s002.zip › Data_S1_AerialSurvey_PM/README_Metadata_AerialSurvey_PM.docx]

**Metadata for aerial survey and participatory mapping data sets**

**File name:** PM_WinterMotorized.shp

**Motorized**

**Description:** Estimated intensity of winter snowmobiling across the study area collected from participatory mapping. Estimates represent the average daily number of recreationists within each area. In areas where participatory mapping participants indicated differing numbers of recreationists in the same place, the highest estimate has been used. Participatory mapping was conducted by Yellowstone to Yukon Conservation Initiative from 2020 – 2021 and included interviews with recreation experts (e.g., park rangers, recreation group, trail users, lodge and campground owners). Participants were asked to identify on a map the areas with recreation, the types of activities, and estimated average number of people per day recreating in these areas from 2017 to 2019.

**File Type:** Shapefile feature class

**Geometry:** Polygon

**Spatial Reference System:** NAD 1983 UTM Zone 11, Transverse Mercator, EPSG: 26911

**Temporal extent:** Winter (snow on ground), 2017 – 2019.

**Field Dictionary**

| **Field name** | **Description** |
| --- | --- |
| FID | Unique identifier for each object in the table. |
| Shape | Geometry (polygon, line, point) |
| Intensity | The average daily number of people recreating in an area, classified into four bins.  0: recreationists present, but no estimate provided.  1: 0 – 10 recreationists.  2: 11 – 50 recreationists.  3: > 50 recreationists. |
| Shape_Area | The area (m^2^) of each polygon. |

**File name:** PM_SummerMotorized.shp

**Description:** Estimated intensity of summer motorized recreation (off-highway vehicles) in the Ghost Public Land Use Zone, Alberta, collected from participatory mapping. Estimates represent the average daily number of recreationists within each area. In areas where participatory mapping participants indicated differing numbers of recreationists in the same place, the highest estimate has been used. Participatory mapping was conducted by Yellowstone to Yukon Conservation Initiative from 2020 – 2021 and included interviews with recreation experts (e.g., park rangers, recreation group, trail users, lodge and campground owners). Participants were asked to identify on a map the areas with recreation, the types of activities, and estimated average number of people per day recreating in these areas from 2017 to 2019.

**File Type:** Shapefile feature class

**Geometry:** Polygon

**Spatial Reference System:** NAD 83 UTM Zone 11, Transverse Mercator, EPSG: 26911

**Temporal extent:** Summer (no snow on ground), 2017 – 2019.

**Field Dictionary**

| **Field name** | **Description** |
| --- | --- |
| FID | Unique identifier for each object in the table. |
| Shape | Feature type (polygon, line, point). |
| Intensity | The average daily number of people recreating in an area, classified into four bins.  0: recreationists present, but no estimate provided.  1: 0 – 10 recreationists.  2: 11 – 50 recreationists.  3: > 50 recreationists. |
| Shape_Area | The area (m^2^) of each polygon. |

**File name:** AerialSurvey_WinterMotorized.tif

**Description:** Percent cover of winter motorized recreation tracks (e.g., snowmobiling tracks) from aerial surveys near the population centers of Golden and Revelstoke in the Kootenay Mountains, British Columbia, Canada. Two rounds of surveys were completed in a helicopter between February and April 2022, with each survey occurring at least a month apart. This dataset represents the combined survey data processed into 100m x 100m cell grids. For cells sampled across the two surveys, the highest footprint value was used.

**File Type:** Raster

**Spatial Reference System:** NAD 83 UTM Zone 11, Transverse Mercator, EPSG: 26911

**Temporal extent:** February – April, 2022.

**Spatial Resolution:** 100m x 100m

**Field Dictionary**

| **Field name** | **Description** |
| --- | --- |
| OID | Unique identifier for each row in table. |
| Footprint | The percent of the observation window covered by recreation tracks (i.e., the footprint), classified into six bins.  0: no recreation  10: < 10%  25: 10 – 25%  50: 26 – 50%  75: 51 – 75%  76: 76 – 100% |
| Count | Number of cells. |
