# Supplementary figures and images for: Advancements in monitoring: a comparison of traditional and application-based tools for measuring outdoor recreation
Source: PeerJ. 2024 Sep 10;12:e17744. doi: 10.7717/peerj.17744 (PMC11397128; doi:10.7717/peerj.17744)

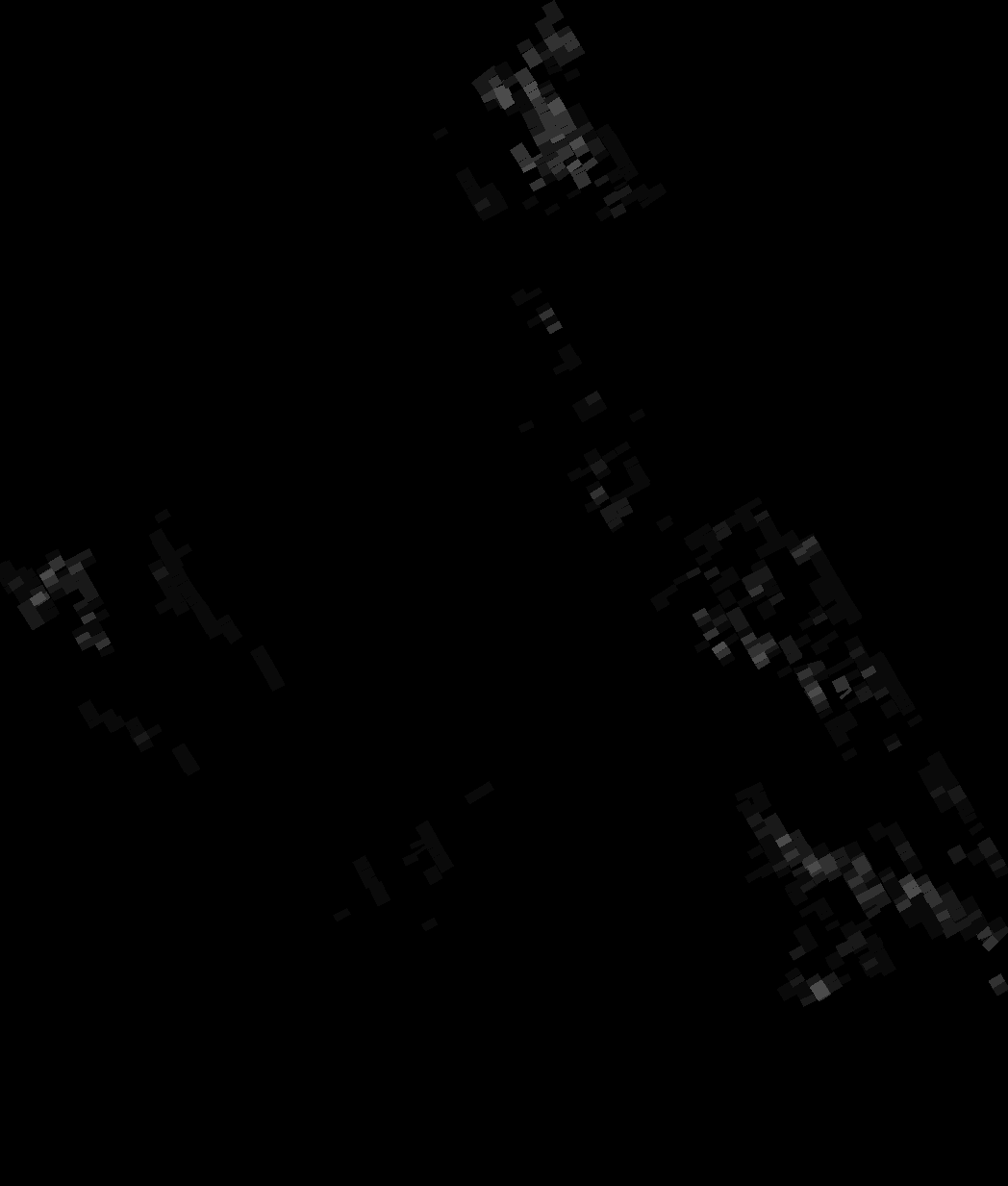

Supplement: Supplemental Information 2 [file peerj-12-17744-s002.zip › Data_S1_AerialSurvey_PM/AerialSurvey_WinterMotorized.tif]

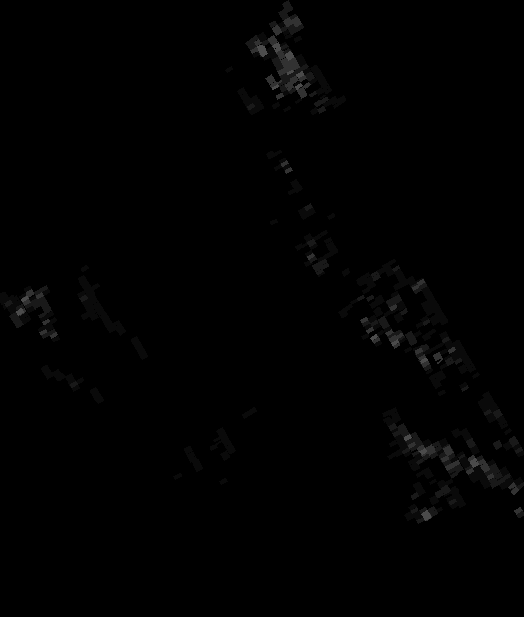

Supplement: Supplemental Information 2 [file peerj-12-17744-s002.zip › Data_S1_AerialSurvey_PM/AerialSurvey_WinterMotorized.tif.ovr]

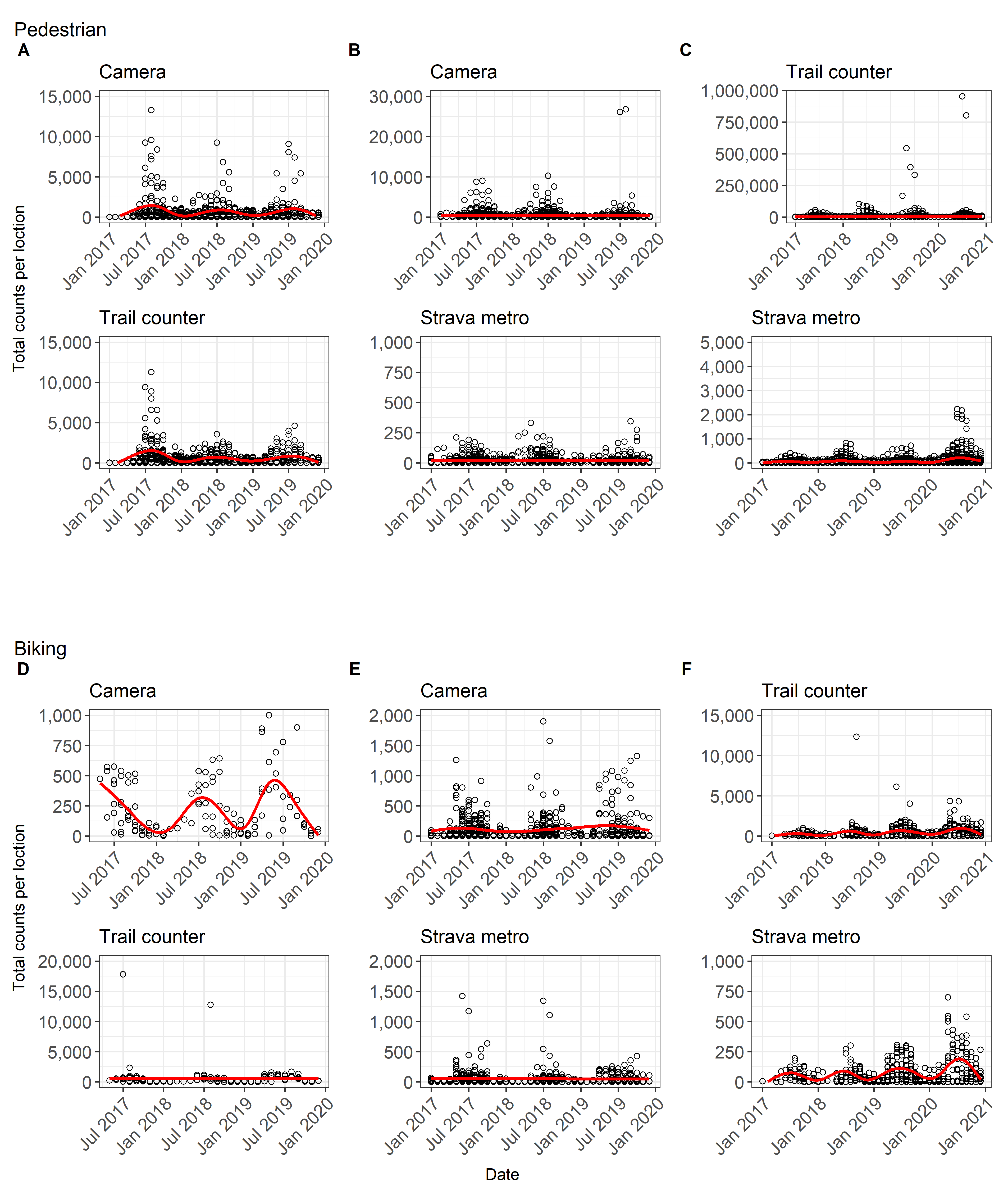

Supplement: Supplemental Information 6 — Each panel represents monthly pedestrian counts between spatially matched locations of (A) cameras and counters, (B) cameras and Strava Metro, (C) counters and Strava Metro, and monthly biking counts from spatially matched locations of (D) cameras and counters, (E) cameras and Strava Metro and (F) counter and Strava Metro from 2017 –2020. The red line represents a trendline generated with generalized additive models. [file peerj-12-17744-s006.png]

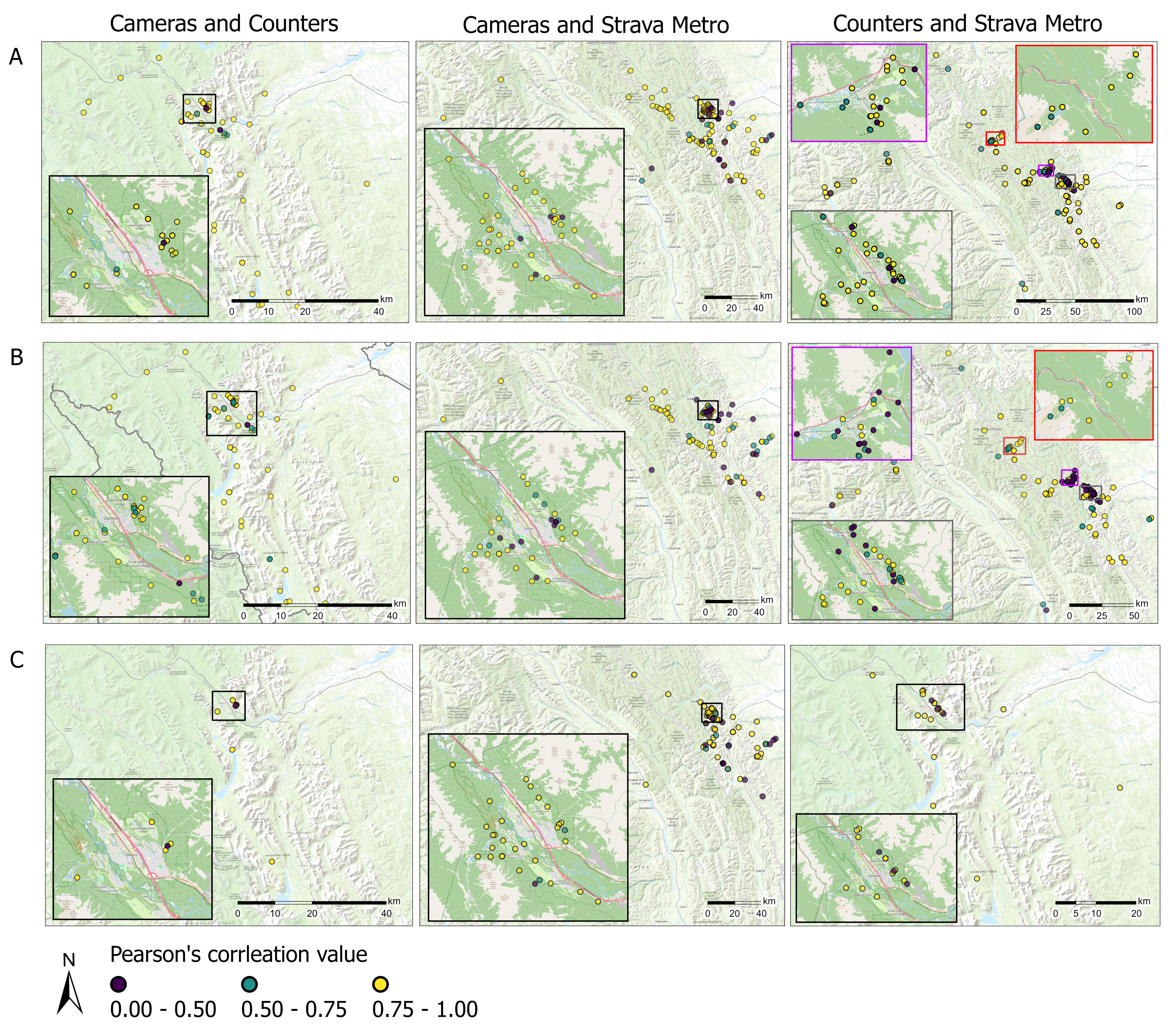

Supplement: Supplemental Information 7 — First column compares counts from cameras and counters; second column compares counts from cameras and Strava Metro; third column compares counts from counters and Strava Metro. Base map source: Esri, HERE, Garmin, Intermap, increment P Corp., GEBCO, USGS, FAO, NPS, NRCAN, GeoBase, IGN, Kadaster NL, Ordnance Survey, Esri Japan, METI, Esri China (Hong Kong), ©OpenStreetMap contributors, and the GIS User Community. [file peerj-12-17744-s007.png]

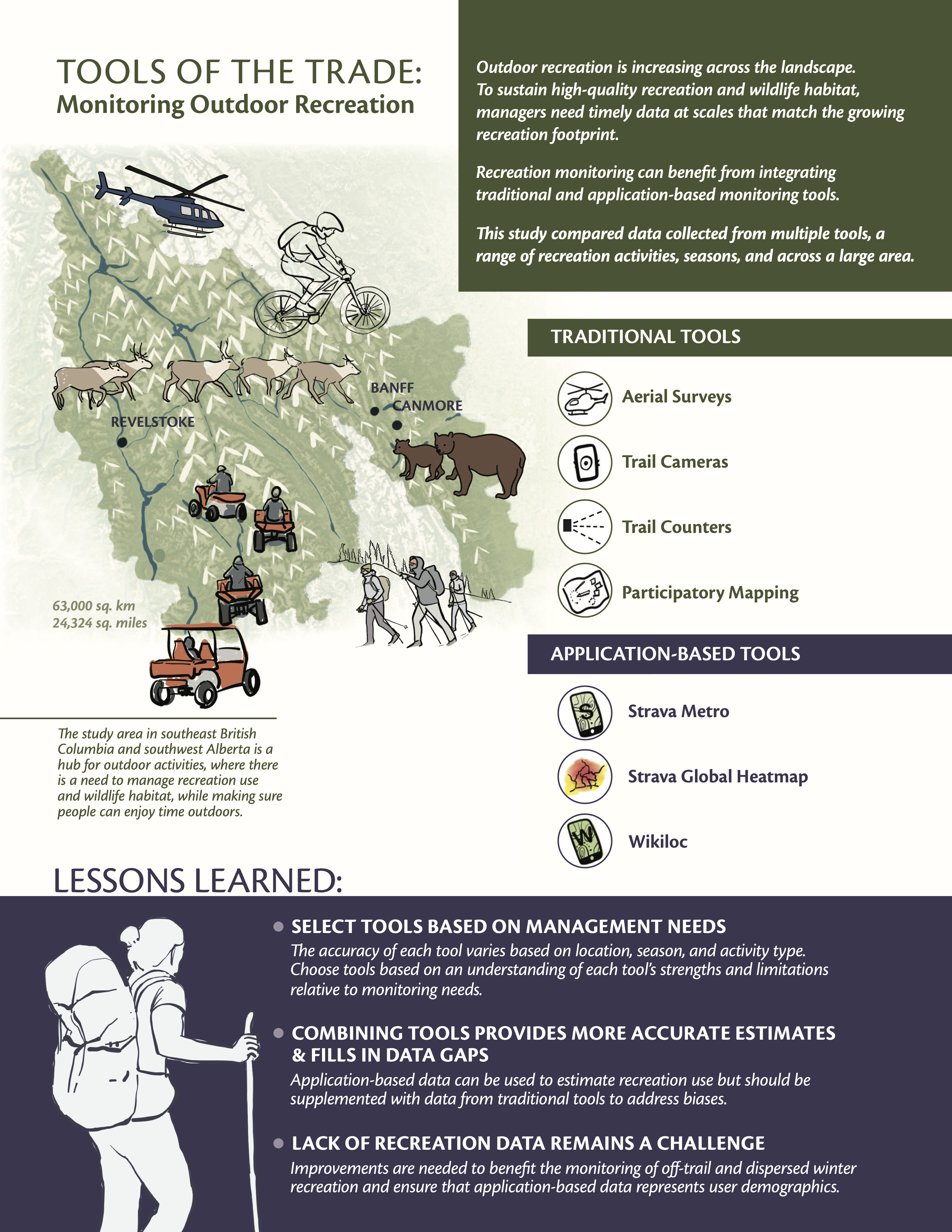

Supplement: Supplemental Information 8 — It also includes lands within Treaties 6, 7, and 8 and districts 1, 3 and 4 of the Otipemiskiwak Métis Government of Alberta. Credits: Loosen Studio (loosenstudio.net). [file peerj-12-17744-s008.png]
