# Supplementary material for: Advancements in monitoring: a comparison of traditional and application-based tools for measuring outdoor recreation
Source: PeerJ. 2024 Sep 10;12:e17744. doi: 10.7717/peerj.17744 (PMC11397128; doi:10.7717/peerj.17744)
Supplement: Supplemental Information 3 — Cameras and counters were matched if devices were within 200 m of each other along a trail and had data on the same day. Cameras and counters were matched to Strava Metro segments within 30 m of devices and had data on the same day. [file peerj-12-17744-s003.pdf]

Table S1. Number of pairwise combinations of camera, counter, and Strava Metro spatially matched locations for all recreation activities, biking, and pedestrian (hike, run, walk). Cameras and counters were matched if devices were within 200 m of each other along a trail and had data on the same day. Cameras and counters were matched to Strava Metro segments within 30 m of devices and had data on the same day.

**Activity type**	**Number of spatially matched locations**		
	Camera and counter	Camera and Strava Metro	Counter and Strava Metro
All activities	58	156	189
Pedestrian	60	157	184
Biking	11	102	36
