# Supplementary material for: Advancements in monitoring: a comparison of traditional and application-based tools for measuring outdoor recreation
Source: PeerJ. 2024 Sep 10;12:e17744. doi: 10.7717/peerj.17744 (PMC11397128; doi:10.7717/peerj.17744)
Supplement: Supplemental Information 4 [file peerj-12-17744-s004.pdf]

**Table S2. Comparison of the select monitoring tools to measure motorized (M) and non-motorized (NM) winter and summer outdoor recreation, including key limitations and advantages for each tool (does not represent an exhaustive list).**

| Data or tool characteristic | Traditional tools | | | | Application-based tools | | |
|---|---|---|---|---|---|---|---|
| | Trail counter | Camera trap | Aerial survey | Participatory mapping | Strava Metro | Strava Heatmap | Wikiloc |
| **Counts of humans or GPS tracks** | Yes[1] | Yes | Yes | No | Yes[2] | No | Yes |
| **Spatial resolution** | High | High | Low to High[3] | Low to High[3] | High | High | High |
| **Temporal resolution** | High | High | Low to High | Low to High[3] | High[4] | Low[4,5] | High[4] |
| **Information on activity type** | Depends[6] | Yes | Yes[3] | Yes[3] | Yes[7] | Yes[8] | Yes |
| **Motorized (M) or non-motorized (NM)** | M and NM | M and NM | M and NM | M and NM | NM[7] | NM[8] | M and NM |
| **Off trail use** | No | No[9] | Yes | Yes | No[10] | Yes[11] | Yes |
| **Data processing burden** | Low | Low to High[12] | Low | Low to High[3] | Low to High[3] | Low to High[3] | Low to High[3] |
| **Consideration for data privacy** | No | Yes | No | Yes[13] | Yes | Yes | Yes |
| **User-group bias** | No | No | No | No | Yes[14] | Yes[14] | Yes[14] |

**Table S2. Continued.**

| Data or tool characteristic | Traditional tools | | | | Application-based tools | | |
|---|---|---|---|---|---|---|---|
| | Trail counter | Camera trap | Aerial survey | Participatory mapping | Strava Metro | Strava Heatmap | Wikiloc |
| **Advantages** | many references for setting up counters, processes and analyzing data | many references for setting up cameras, processing and analyzing data | continuous coverage of large areas; can capture photos and video; qualitative observations | qualitative information; can design methodology to capture areas of interests and level of detail | includes direction of travel | global coverage; includes more activities than Strava Metro (includes winter and water[8]) | targets outdoor users in more remote locations; many recreation activities; can include photos and trip descriptions |
| **Limitations** | cannot differentiate between people, moving plants or animals, repeated counting if people stop in front of device; data error from environmental conditions and how people align themselves along trail; difficult to capture off-trail use or trail networks with numerous entry points; malfunction and tampering of device | difficult to capture off-trail use or trail networks with numerous entry points; malfunction and tampering of device | costly and weather dependent that can limit the spatial and temporal coverage; potential disturbance to recreationist and wildlife; potential observer biases; safety concerns | sensitive to cognitive bias and misrepresentation of recreation on map; can be labor intensive to facilitate interviews and digitize paper maps or questionnaires; for large areas may have discontinuous coverage or incomplete information | biking and pedestrian recreation only; represents app-users only | does not provide real counts of recreation; only available at annual scale (not daily or monthly); heatmap values are comparable locally; can't separate recreation activities (i.e., ski mountaineering, cross-country skiing, ice skating, etc.[8]); represents app users-only | requires manually downloading each track; represents app-users only |

[1] Cannot differentiate between individuals or groups, such that counts represent estimates not absolute counts. However, placement of counters along narrow trail sections can capture areas where recreationists are more likely to be traveling single-file.

[2] Only includes activity counts (over 5 users) on trails in OpenStreet Maps.

[3] Can vary depending on context and sampling design.

[4] Growing popularity with some recreation groups over time that need to be accounted for in temporal analyses.

[5] Only annual heatmaps are available (i.e., no daily or monthly heat maps are available).

[6] Some devices can specifically count bikes and cars.

[7] Includes only pedestrian (hike, walk, run) and biking activities.

[8] At the time of this analysis, Strava Global Heatmap data was aggregated into four broad recreation activity categories: water, winter, pedestrian (hike, walk run), and bike. Since then, Strava Global Heatmap has been updated with more specific categories of recreation activities (https://www.strava.com/heatmap).

[9] Typically cameras placed to monitor recreation and wildlife trails or wildlife attractants (e.g., tree rubs for grizzly bears).

[10] All trails segments snapped to OpenStreetMaps street and trail segment.

[11] Strava Global Heatmap covers open bodies of water (i.e., rivers, lakes, reservoirs, canals)

[12] Advancements of automatic image processing software can speed up data processing.

[13] For participatory mapping participants.

[14] Unequal distribution of users, with some users contributing more data, more frequently
