# Supplementary material for: Advancements in monitoring: a comparison of traditional and application-based tools for measuring outdoor recreation
Source: PeerJ. 2024 Sep 10;12:e17744. doi: 10.7717/peerj.17744 (PMC11397128; doi:10.7717/peerj.17744)
Supplement: Supplemental Information 5 [file peerj-12-17744-s005.pdf]

**Supplemental Data Description S1**

**Recreation data sources: data collection methods**

***Camera trap***

We compiled camera trap data shared from Parks Canada and Alberta Parks remote camera trap networks (Steenweg et al., 2016; Stewart et al., 2016; Whittington, Low & Hunt 2019). Recreation included motorized (motorbike, quad, snowmobile, vehicle) and non-motorized (angler, bike, climb, electric bike, fat bike, hike, hunt, run, ski, skijorer, snowshoe) activities. We categorized activity types into three categories: all activities (all motorized and non-motorized activities), pedestrian (angler, climb, hike, hunt, run, ski, skijorer, snowshoe) and biking (bike, electric bike, fat bike). A summary of camera sampling design and image processing approaches for each project are detailed below.

*Steenweg R, Whittington J, Hebblewhite M, Forshner A, Johnston B, Petersen D, Shepherd B, Lukacs PM. 2016. Camera-based occupancy monitoring at large scales: power to detect trends in grizzly bears across the Canadian Rockies. Biological Conservation 201:192–200. DOI: 10.1016/j.biocon.2016.06.020.*

Steenweg et al. (2016) monitored changes in the occupancy of grizzly bears (*Ursus arctos*) using camera-based occupancy models across the Canadian Rockies. They deployed 183 cameras per a 10 x 10 km cell across the Canadian Rockies study area including Jasper, Banff, Yoho, Kootenay and Waterton Lakes National Parks in 2012. They used mainly covert motion-trigger cameras (Hyperfire and Rapidfire models; Reconyx, Holmen, Wisconsin) and some visible glow cameras (Silent-image, Reconyx). Sites were selected to where animals were likely to travel based on

topography, confluence of wildlife trails and grizzly bear rub trees. Cameras were set at waist height, pointed slightly downwards, and set to take five images per movement trigger with no delay between triggers. No bait was used and cameras were serviced 3 to 4 times a year. The Timelapse2 software (Greenberg and Godin, 2015) was used to classify images into species, sex and age classes. Picture events (i.e., independent detection events) were identified as images of grizzly bears at least 5 minutes apart.

*Stewart FEC, Heim NA, Clevenger AP, Paczkowski J, Volpe JP, Fisher JT. 2016. Wolverine behavior varies spatially with anthropogenic footprint: implications for conservation and inferences about declines. Ecology and Evolution 6:1493–1503. DOI: 10.1002/ece3.1921.*

Stewart et al. (2016) used camera traps to examine spatial patterns in the behavior of wolverine (*Gulo gulo)* across three study areas in the Rocky Mountains of Alberta, Canada. They deployed 164 Reconyx digital cameras (models RM30, PM30, PC900; Reconyx, Holmen, Wisconsin) in a systematic design composed of 12 x 12 cell grids between December and April, 2017 – 2013. Sites were baited. Cameras were programmed to take 5 photographs at 1 second intervals, repeated at each detected movement. We received this camera trap data categorized in a table, displaying for each camera image, the species, the total number of individuals in an image, and the date and time.

*Whittington J, Low P, Hunt B. 2019. Temporal road closures improve habitat quality for wildlife. Scientific Reports 9:3772. DOI: 10.1038/s41598-019-40581-y.*

Whittington et al. (2019) assessed the response of nine mammal species to temporal road closures in Banff National Park, Canada. They used a combination of remote cameras, road

surveys and movement data from grizzly bear (*Ursus arctos*) global position system collar data. Motion triggered cameras (Reconyc Hyperfire PC900 Professional cameras, Holmen, Wisconsin) were part of Parks Canada broader remote camera network. Data was selected from 10 cameras along the Bow Valley Parkway and 54 reference cameras (18 on trails and 36 on highway crossing structures) from 2014 – 2017. Independent detection events were classified as whether or not at least one wildlife species was detected for each camera sampling day and hour.

***Trail counters***

We compiled data from passive infrared TRAFx trail or vehicle counters (TRAFx Research Ltd., Canmore, Alberta, Canada) shared with us from the government of Alberta, Parks Canada, Nature Conservancy of Canada (NCC) and Recreation Sites and Trails British Columbia (RSTBC). Trail counters were deployed to monitor human recreation activity. In Kananaskis Country, Alberta, Canada, a selection of these trail counters were paired with camera traps to examine differences in counts between the two tools to determine if the counters were installed in suitable locations and collected accurate data (Alberta Parks, *personal communications*).

***Participatory mapping (PM)***

Participants were selected from a list of recreation experts (i.e. park rangers, recreation groups, trail users, lodge and campground owners) known to Yellowstone to Yukon Conservation Initiative (Y2Y; y2y.net). Participants were recruited by email with an explanation of the Y2Y recreation ecology research project (Fig. S1) and the areas where the project was interested in understanding human recreation use patterns (Fig. S2).

From 2020 – 2021, meetings with 36 participants were conducted online over Zoom (Zoom Video Communications, Inc. 2020, San Jose, California, United States) and were recorded with the permission of participants. We started with a brief introductory explaining the research project and study area, outlined how we were going to proceed with the mapping, and asked if there were any questions or concerns. Participants were asked to load the web-based platform Gaia GPS (www.gaiagps.com) and to share their screen displaying an interactive map. They were asked to use the interactive map to show areas they were familiar with and to explain what the average recreational use of that area was between 2017 – 2019.  We asked if they could estimate daily use from 1–10 people, 11–50 and over 50 people a day and if they could specify what activity occurred in the area. If participants could only indicate if recreation was present or use within other years, we collected that information as well.

Recordings of meetings were watched and areas where participants described as having recreation use were digitized in ArcMap (ArcMap Version 10.8.2, ESRI, Redlands California) or QGIS (QGIS Development Team, QGIS Geographical Information System, Open Source Geospatial Foundation, http://www.qgis.org) in points, lines or polygons with the activity type and use intensity was added to the attribute table. There was variation in the spatial resolution of the polygons and where participants indicated there to be recreation; some participants provided estimates of the number of recreationists on a specific trail or camping area, while other participants provided recreationists estimates for a valley, land use zone or a large portion of our study area. Similarly, how participants provided estimates of recreationists varied. Some participants included information on the number of recreations over a weekend or throughout the week, sometime differentiate between seasons (i.e., summer or winter) or did not specify a time

period. Where appropriate, estimates were adjusted to reflect estimated number of recreationists per day.

In ArcGIS we classified all polygons and polylines of recreation use by activity type and season – summer (no snow on ground), winter (snow on ground), spring (snow only in alpine), fall (hunting season) – and a unique identification number was added to each individual polygon or polyline to identify the feature. A fishnet of grid cells was created to overlap each activity type and the unique identification number of each underlying polygon or polyline was extracted to each grid cell. Recreation count estimates were binned into three categories (0–10; 11–50; > 51 recreationists per day) and the maximum bin values was joined to the grid cells.

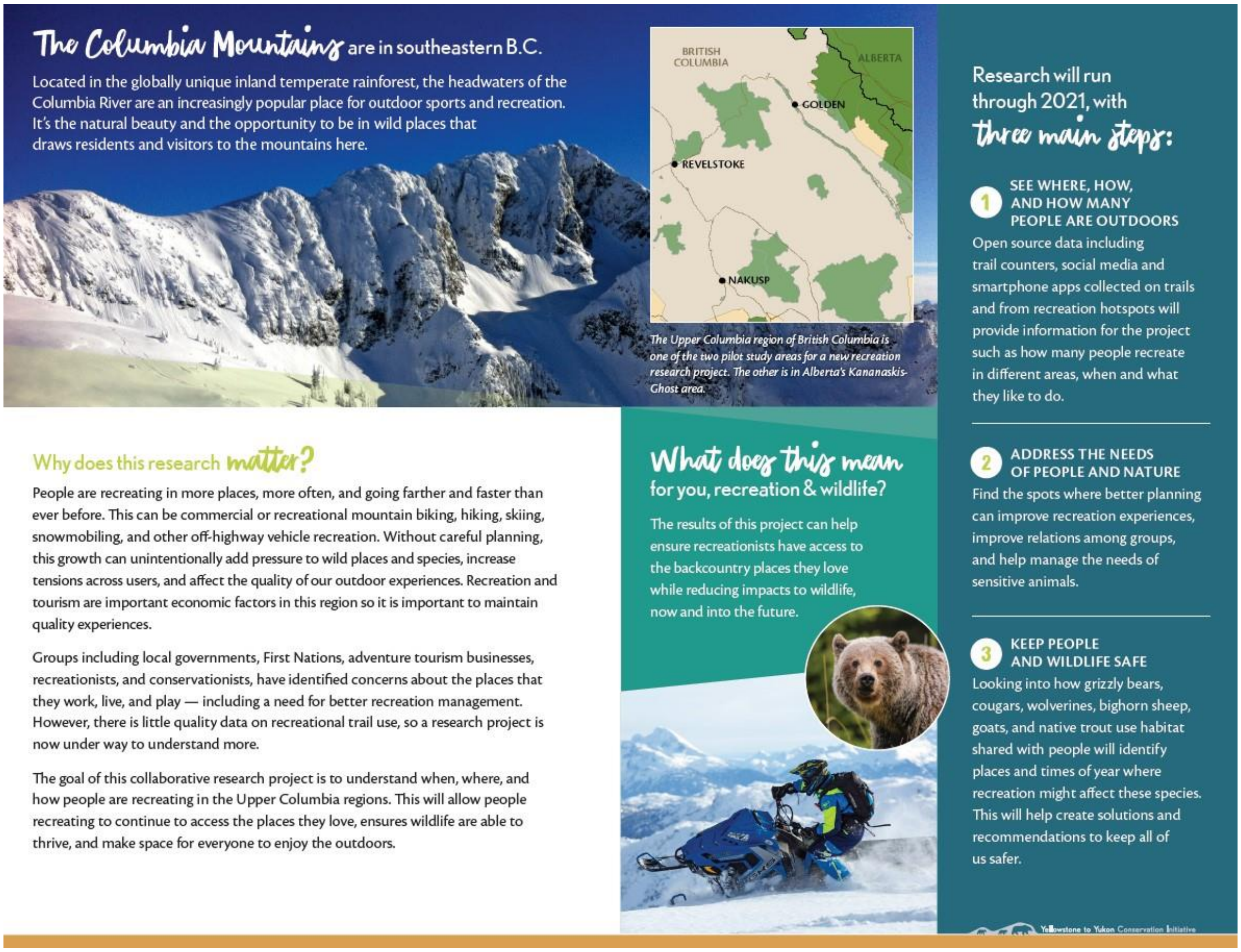


**Figure S1. Informational pamphlet sent via email to recruit volunteers in participatory mapping research to support the Yellowstone to Yukon Conservation Initiative recreation ecology project**. The image, and associated context, is out of date and reflects the early stages of the multi-year research project. Source credit: Yellowstone to Yukon Conservation Initiative.

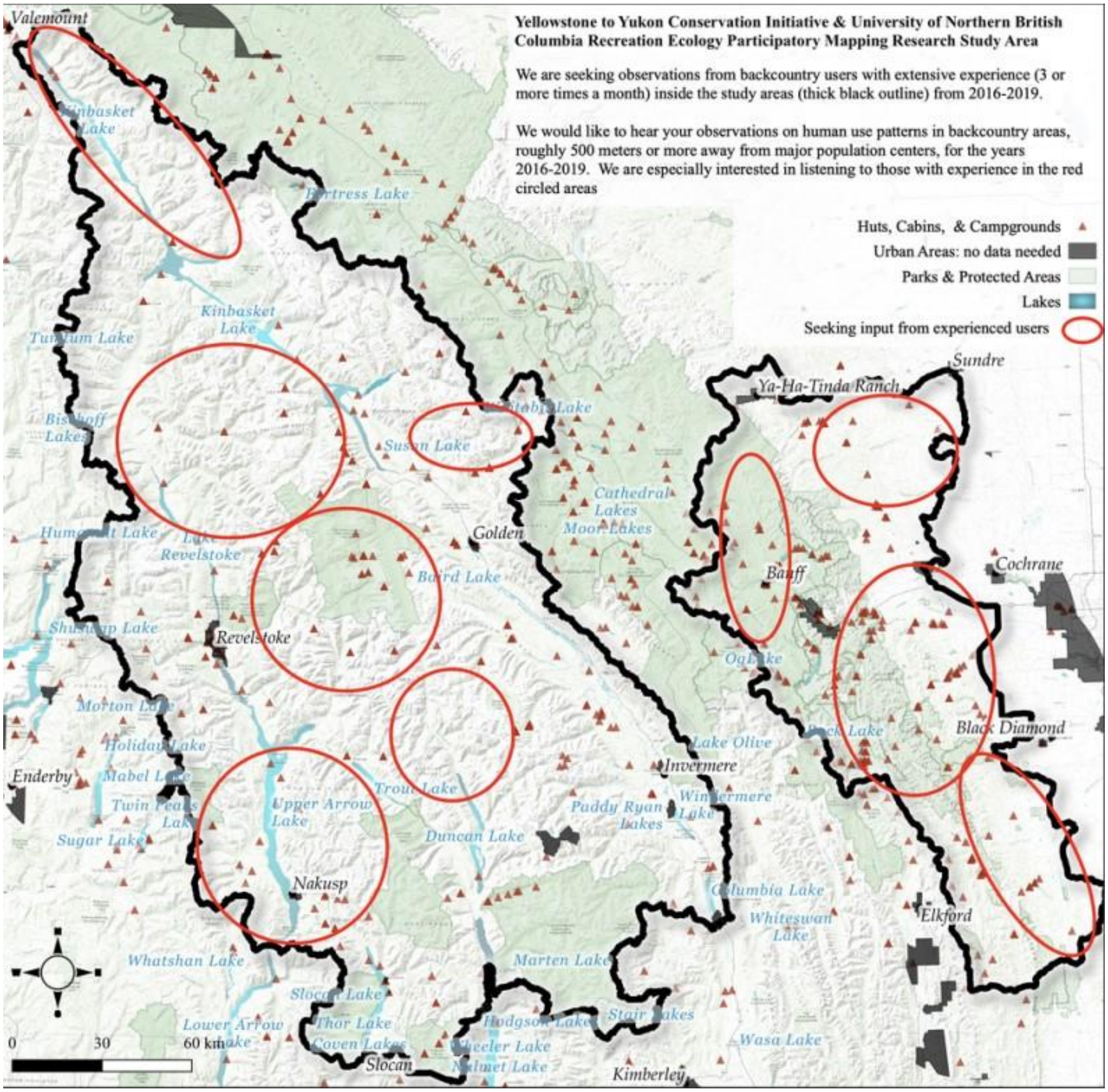


**Figure S2. Image of areas we were interested in collecting participatory mapping data sent via email to recruit volunteers to support the Yellowstone to Yukon Conservation Initiative recreation ecology project.** The image reflects the early stages of the multi-year research project. While the image indicates that we were interested in human use patterns for the years 2016 – 2017, we actually asked for information for the years 2017 – 2019. The study area in the image reflects the original study area boundary that was changed for latter years of the multi-year project. Base map source: Esri, USGS, NOAA, Garmin, NPS.

### *Aerial surveys*

We conducted aerial surveys (Heinemeyer et al., 2019) in three focal areas in Alberta and British Columbia: i) the Kananaskis area south of Canmore, ii) the Upper Columbia between Golden and Revelstoke in British Columbia (BC), and iii) the Lake Louise section of Banff National Park, Yoho and Kootenay National Parks, Canada (Fig. S3). We selected flight areas in consultation with provincial and federal biologists, conservation officers and public safety specialists. A grid of 1.5 x 1.5 km cells overlaid the flight areas (Heinemeyer et al., 2019). Flight lines followed every other grid cell, resulting in transects spaced 3 km apart (Fig. S3). To survey, we flew the transect lines in a helicopter at an altitude of 305 m (1,000 feet) at a speed of 145 km/hr (90 miles/hour; Heinemeyer et al., 2019). In the rear of the helicopter, two primary observers sat on the left and right sides, each responsible for searching for snowmobiling, skiing (heli-skiing, backcountry skiing and cat-skiing) tracks. Observers recorded the recreation activity type, the percent of the observation window covered by recreation tracks (i.e., the footprint; none, 1–10 %, 10–25%, 26–50%, 51–75% and 76–100%), and track age (new – tracks were set after latest snowfall; old – tracks are snow covered). GPS waypoints were collected on handheld units every 20 seconds, during which observers scanned a 1.5 x 1.5 km area on each side of the helicopter. This defined the observation window. The survey leader ensured the helicopter followed each flight line and called out waypoint numbers to indicate to the observers when a new observation window had started or ended (Heinemeyer et al., 2019). Permit numbers were Alberta Parks (#22-024) and Parks Canada (#LL-2022-41098; RAP #KO22-001).

To match point observations to a viewing window, we created irregular grids extending out 1.5 km and forwards from the waypoint at which the observation window started to the next sequential waypoint. If there was a change in bearing (e.g. due to going off-course to avoid a

peak, strong winds), the irregular grids were always perpendicular to the flight line (i.e., line between two waypoints; Fig. S4).

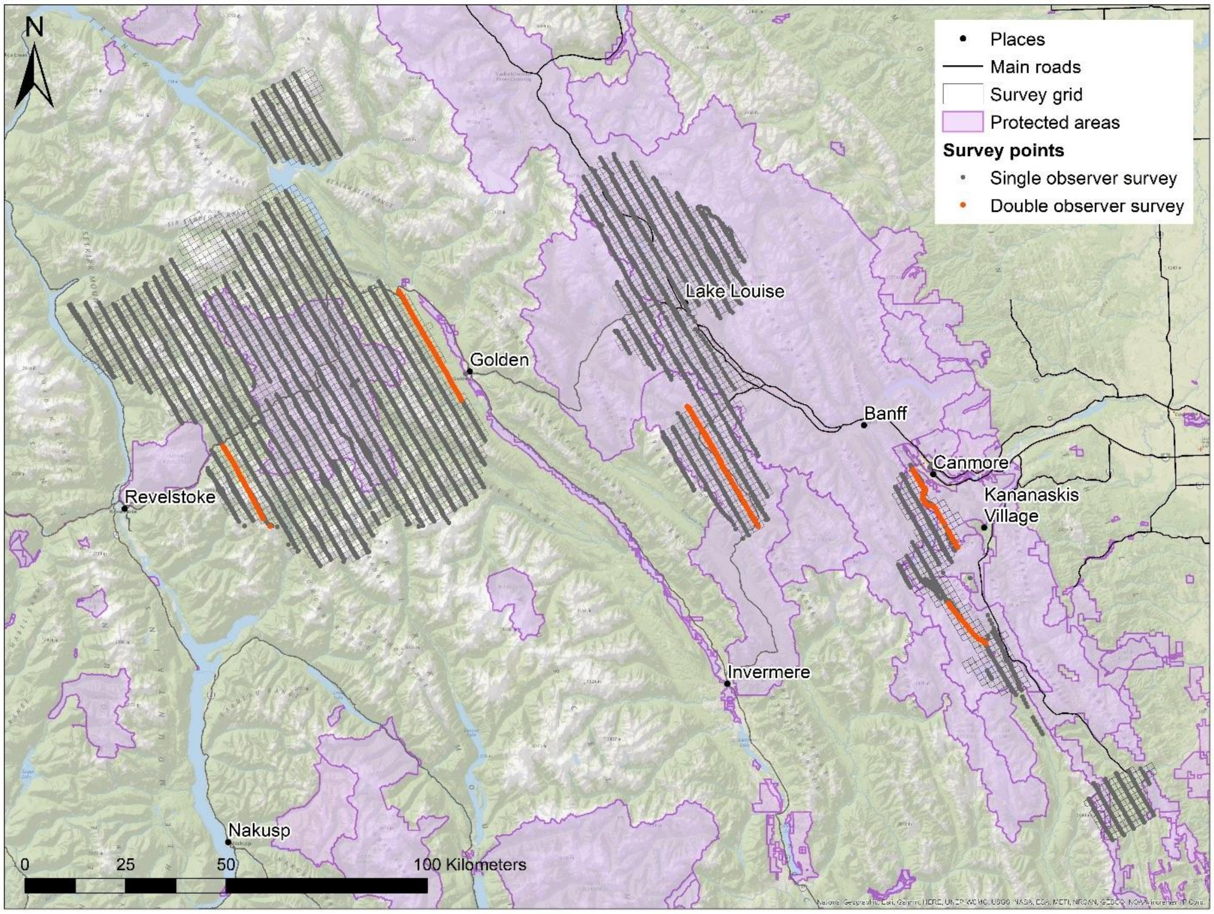


**Figure S3. Map of winter recreation aerial surveys in southeastern British Columbia and southwestern Alberta, Canada, in winter 2022**. Grey dots represent single observer survey waypoints. Base map source: National Geographic, Esri, Garmin, HERE, UNEP-WCMC, USGS, NASA, ESA, METI, NRCAN, GEBCO, increment P Corp.

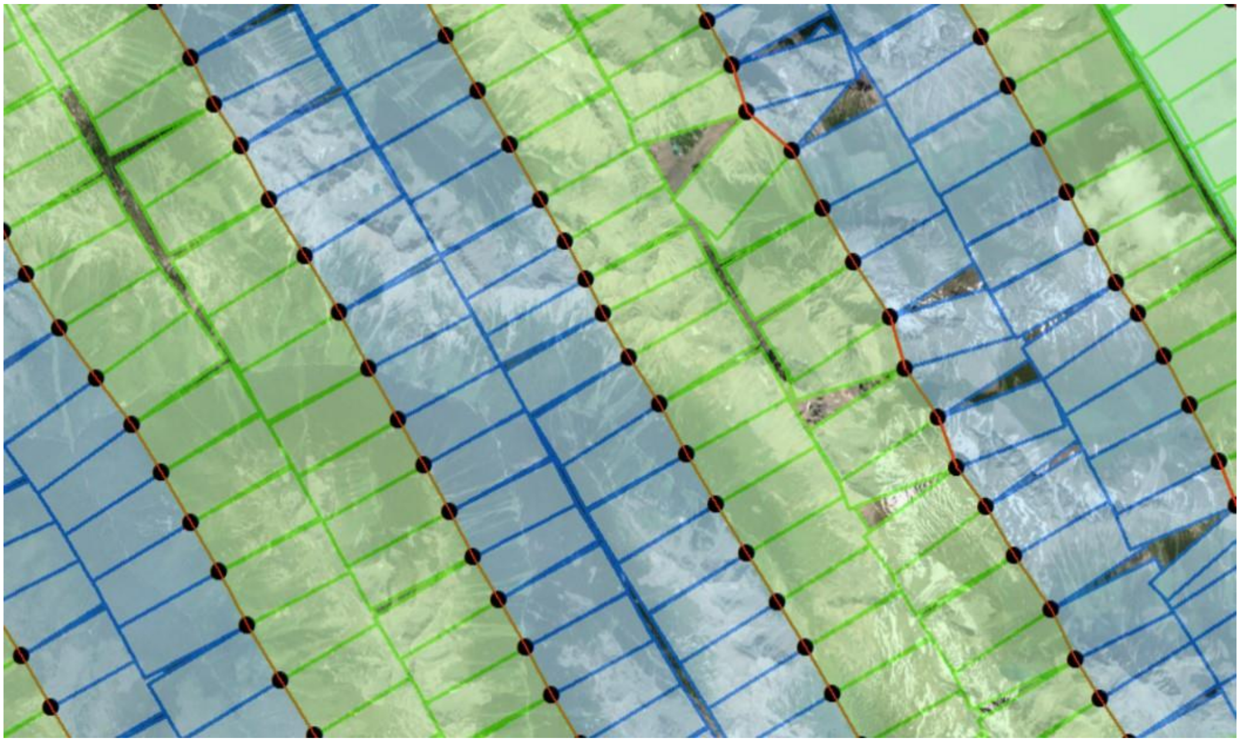

**Figure S4. Sample of an irregular grid for winter recreation aerial surveys.** Dots represent location of waypoints taken from the helicopter and the red line is the flight path of the helicopter. The blue boxes are the left-side observation window and green are right side observations. The grid length perpendicular to the red line is 1.5 km, representing how far out from the helicopter observers scanned for recreation tracks, while the width of the box was determined by the location of the following waypoint. Base map source: Esri, Maxar, Earthstar Geographics, and the GIS User Community.

***Strava Global Heatmap***

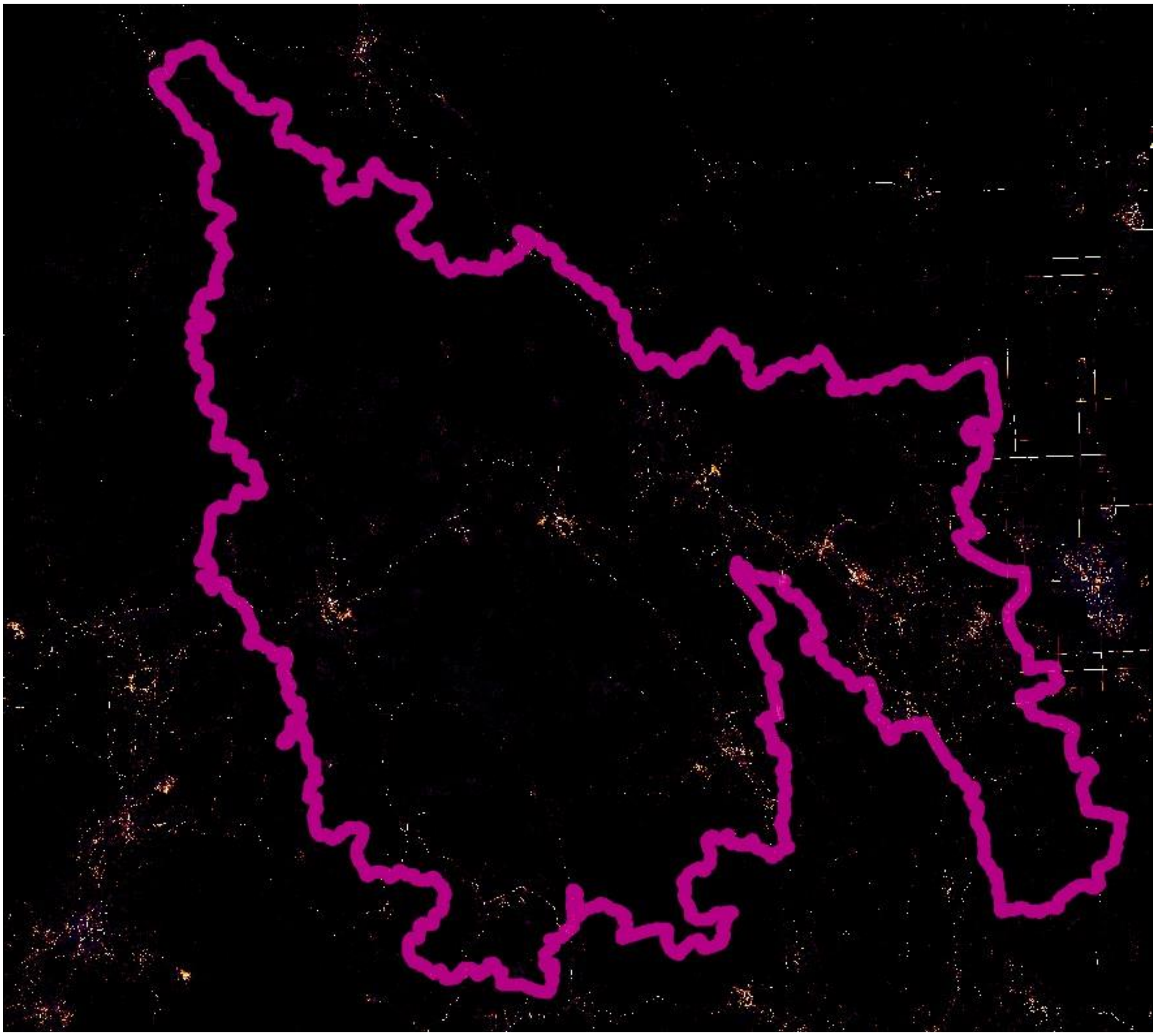

**Figure S5. Strava Heatmap from 2016 – 2017, with study area border denoted in pink.** Strava Heatmap represents cumulative biking, pedestrian, winter and water recreation activities from Strava users. Source: Strava Global Heatmap (https://www.strava.com/maps).

## References

Greenberg, S, Godin, T, 2015. A tool supporting the extraction of angling effort data from remote camera images. Fisheries 40, 276–287. DOI: 10.1080/03632415.2015.1038380.

Heinemeyer K, Squires J, Hebblewhite M, O'Keefe JJ, Holbrook JD, Copeland J. 2019. Wolverines in winter: indirect habitat loss and functional responses to backcountry recreation. Ecosphere 10:e02611. DOI: https://doi.org/10.1002/ecs2.2611.

Steenweg R, Whittington J, Hebblewhite M, Forshner A, Johnston B, Petersen D, Shepherd B, Lukacs PM. 2016. Camera-based occupancy monitoring at large scales: power to detect trends in grizzly bears across the Canadian Rockies. Biological Conservation 201:192–200. DOI: 10.1016/j.biocon.2016.06.020.

Stewart FEC, Heim NA, Clevenger AP, Paczkowski J, Volpe JP, Fisher JT. 2016. Wolverine behavior varies spatially with anthropogenic footprint: implications for conservation and inferences about declines. Ecology and Evolution 6:1493–1503. DOI: 10.1002/ece3.1921.

Whittington J, Low P, Hunt B. 2019. Temporal road closures improve habitat quality for wildlife. Scientific Reports 9:3772. DOI: 10.1038/s41598-019-40581-y.
